# Exotic mitotic mechanisms

**DOI:** 10.1098/rsob.120140

**Published:** 2012-12

**Authors:** Hauke Drechsler, Andrew D. McAinsh

**Affiliations:** Centre for Mechanochemical Cell Biology, Division of Biomedical Cell Biology, Warwick Medical School, University of Warwick, Coventry CV4 7AL, UK

**Keywords:** kinetochore, nuclear pore complex, mitosis, spindle, chromosome

## Abstract

The emergence of eukaryotes around two billion years ago provided new challenges for the chromosome segregation machineries: the physical separation of multiple large and linear chromosomes from the microtubule-organizing centres by the nuclear envelope. In this review, we set out the diverse solutions that eukaryotic cells use to solve this problem, and show how stepping away from ‘mainstream’ mitosis can teach us much about the mechanisms and mechanics that can drive chromosome segregation. We discuss the evidence for a close functional and physical relationship between membranes, nuclear pores and kinetochores in generating the forces necessary for chromosome segregation during mitosis.

## 2. Introduction

**Figure 1.**
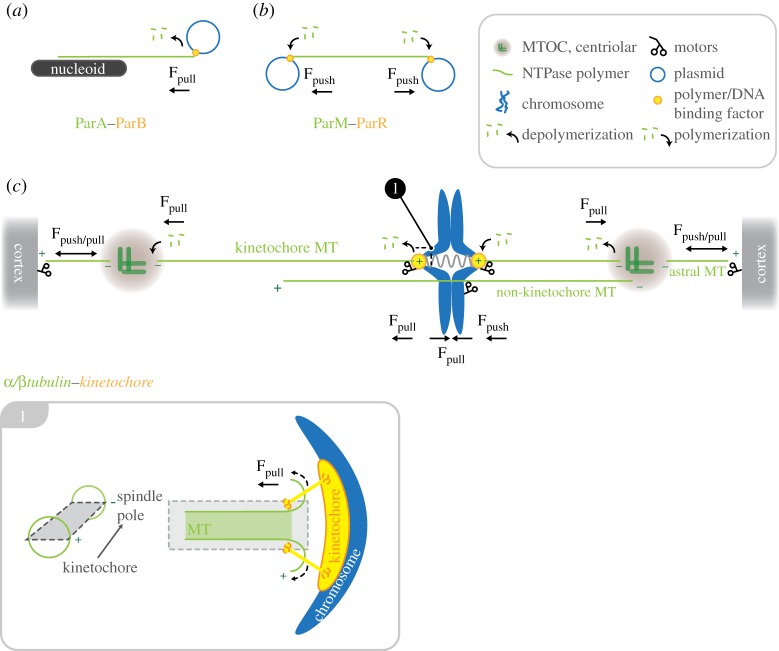
Schematic diagram showing the set-up of the chromosome segregation machinery in bacteria and mammalian cells. The basic requirement for chromosome segregation is a dynamic NTPase polymer (ParA, ParM in prokaryotes or tubulin in eukaryotes; green) that can create pushing (F_push_ in *a,c*) or pulling forces (F_pull_ in *b,c*) by polymerization or depolymerization, respectively. Dynamic NTPase polymers are connected to DNA by centromere binding factor: ParB, ParR in prokaryotes (*a*,*b*) or the kinetochore complex in mammalian cells (*c*) (orange). In contrast to the simplified prokaryote machinery (*a*,*b*), chromosome segregation in eukaryotes is driven by a sum of forces created by tubulin (de)polymerization at kinetochores (plus-end) and depolymerization at spindle poles (minus-ends), in addition to MT-dependent motors bound at kinetochores, chromosome arms, microtubule-organizing centres and the cell cortex. Inset: longitudinal section through a kinetochore-bound microtubule. Curling of kinetochore-MT protofilaments at the plus-end produces pulling forces on the attached kinetochore-chromosome.

The accurate segregation of replicated genomes into daughter cells during cell division is a prerequisite for life in all three domains of life: *Archaea*, bacteria, *Eukaryota*. The segregation process is essentially a mechanochemical problem in that the chromosomes, which have mass, need to be pulled or pushed into daughter cells. All mechanisms known so far can be reduced to two basic components: (i) directional force-generating mechanisms that consume chemical energy provided by hydrolysis of nucleotide triphosphates (NTP), and (ii) an adaptor that physically links this machinery to the carriers of genetic information (chromosomes/plasmids; figure 1). In prokaryotes, the task of DNA segregation is simplified, because the circular chromosome and plasmids are not located within a nucleus. Prokaryotes therefore do not need to do a nuclear division (mitosis). Instead, they partition the duplicated DNA into opposite halves of the cell prior to cell division (binary fission). Basic plasmid partitioning machineries have been identified that involve a centromeric DNA-binding protein and one of three NTPase (for reviews, see [1,2]). The ParM, ParA and TubZ NTPases can form filaments that undergo cycles of nucleotide-dependent polymerization/depolymerization to generate the pulling and/or pushing forces necessary for partitioning (figure 1*a*,*b*). ParM forms filaments that are structurally similar to eukaryotic actin [3], whereas recent work has revealed a third partitioning machine that involves the treadmilling of TubZ-a protein with a tubulin/FtsZ fold [4]. Thus, prokaryotes contain simple DNA-partitioning machines that use proteins resembling the cytoskeletal proteins driving chromosome segregation in eukaryotes. One exception is the segregation of high-copy-number plasmids in prokaryotes, which are thought to use passive diffusion [5,6]. Furthermore, the mechanisms that drive directional chromosome segregation remain elusive [7]. Chromosome segregation in eukaryotes occurs during mitosis and has been under investigation for over 150 years [8], with early work in plant and animal cells, whose chromosomes and their movements could be visualized and described with a light microscope. In this review, we aim to rekindle interest in how natural selection, within eukaryotes, has driven the development of diverse solutions to the task of chromosome segregation. Therefore, we do not provide a comprehensive description of all mitoses that are known, but rather focus on a number of key organisms that exemplify divergence.

## 3. ‘Mainstream’ mitosis

It is necessary to first set out the cell biological transitions and biophysics that underpin ‘mainstream’ mitosis in animal cells. For a comprehensive treatment of this subject, we point the reader to the excellent recent review by McIntosh *et al.* [9]. ‘Mainstream’ mitosis in animal cells is powered by a microtubule-based machine called the mitotic spindle. Microtubules are hollow tubes of 25 nm diameter that assemble from heterodimers of α- and β-tubulin. The head-to-tail assembly of subunits gives rise to a polar polymer with a plus-end and minus-end that differ in their dynamic properties. The mitotic spindle is a bipolar array of microtubules, in which the minus-ends of microtubules are focused at spindle poles, and the plus-ends radiate outwards towards the opposite pole. In animal cells, the primary microtubule-organizing centre (MTOC) is the centrosome, which is located at the spindle pole and consists of two centrioles and the associated pericentriolar material. Non-centrosomal pathways also contribute, and include chromosome- and microtubule-based microtubule nucleation events [10,11]. Spindle microtubules can be separated into those whose plus-ends terminate at kinetochores (K-MTs) and those that remain free in the spindle (non-kinetochore MTs, nK-MTs; also termed inter-polar MTs). nK-MTs have a half-life an order of magnitude shorter than K-MTs [12], but can also form anti-parallel overlaps that are important for spindle bipolarity (figure 1*c*).

K-MT polymerization and depolymerization function as the central motor that drives chromosome movements in mitosis [13] (figure 1*c*, inset 1). Polymerization involves the binding of GTP–tubulin heterodimers to the microtubule end. Subsequent GTP hydrolysis evokes a conformational change in the heterodimer, resulting in energy being stored as strain within the MT lattice. MT polymerization can do work by overcoming Brownian fluctuations, resulting in the generation of pushing forces [14], whereas depolymerization of MTs can also do work because kinetochores have the capacity to grip and maintain attachment to the K-MT plus-end during shrinkage [15]. In addition, the release of lattice strain (see above) causes the bending of protofilaments that could function as a powerstroke (figure 1*c*, inset 1). Kinetochores are thus the eukaryotic equivalent of the centromeric binding component of the prokaryotic partitioning machines, with tubulin being the functional counterpart of the ParA/M/TubZ components. However, unlike these basic prokaryotic two-component systems that use a simple adaptor protein, kinetochores are megadalton-sized protein machines that form attachments to microtubule plus-ends and are assembled from around 150 protein subunits [16–19]. Kinetochores are also pulled polewards, because K-MTs are disassembled at their minus-ends, which are embedded at spindle poles (figure 1*c*). The segregation of sister chromatids towards spindle poles (anaphase A) is thus a composite of subunit removal at the minus- and plus-ends of kinetochore-bound MTs. Completion of anaphase additionally requires spindle elongation (anaphase B), which is driven by microtubule–microtubule sliding within the mid-zone, and the pulling forces exerted by astral–microtubule cortex interactions [20] (figure 1*c*).

In animal cells, disjunction of sister chromatids necessitates the formation of bi-oriented (amphitelic) sister kinetochore attachments; that is, sister kinetochores forming attachments to microtubules emanating from opposite spindle poles, prior to anaphase onset. A sister kinetochore is therefore tasked with not only forming end-on attachment to spindle microtubule plus-ends (capture) in early mitosis, but also forming the selective stabilization of bi-oriented attachments as mitosis progresses. This process involves active error correction mechanisms that destabilize inappropriate attachments (e.g. both sisters bound to a single pole) [21]. In animal cells, these events are coincident with the movement of chromosomes to the spindle equator during prometaphase (congression). Kinetochores can also form attachments to the lattice of microtubules and, through the action of the molecular motor CENP-E, ‘slide’ towards the plus-end of microtubules and the spindle equator [22–24]. In fact, experiments show that congression can occur in the complete absence of end-on attachments, suggesting a more ‘basic’ alignment process that does not involve microtubule dynamics [25]. Finally, kinetochores mediate the spindle assembly checkpoint (SAC), which senses when sister kinetochores are incorrectly attached and generates a soluble signal that inhibits anaphase onset until all sister kinetochores are correctly attached [26,27]. The nature of the signal that the kinetochore senses and how it is satisfied remains unknown, but is likely to reflect the occupancy of MT attachment sites and/or the imposition of tension/force balance across the sister pairs.

## 4. Nuclear envelope breakdown

The evolution of the eukaryotic lineage has, however, presented major new challenges for the chromosome segregation machinery because the genome, which is organized into multiple linear chromosomes, is carried during interphase within a nucleus, whose boundary is a double lipid bilayer called the nuclear envelope (NE). The evolutionary advantage of a nucleus is likely to be the capacity to compartmentalize biochemical processes, such as splicing and transcription, and to maintain the integrity of the genome. The NE contains nuclear pore complexes (NPCs), which are multi-protein (i.e. nucleoporins) assemblies that control the trafficking of molecules between the nucleus and cytoplasm [28]. The problem is that the MTOC, which is necessary for bipolar spindle formation, has to be located in the cytoplasm in order to nucleate the (interphase) microtubule cytoskeleton and organize the assembly of flagella and cilia, whereas the chromosomes are located in the nucleus.

**Figure 2.**
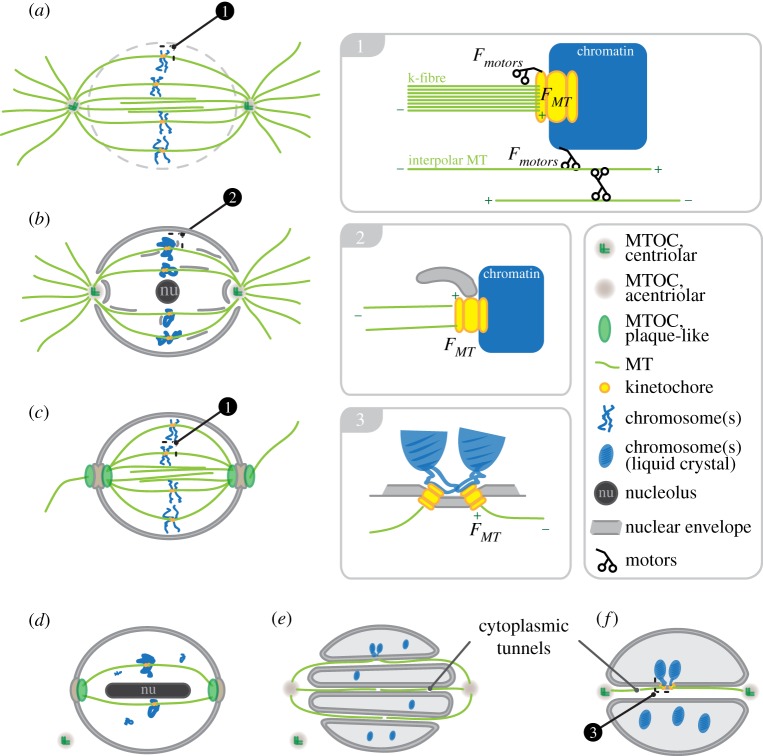
Diverse metaphase spindle set-ups in eukaryotes: (*a*) animals, (*b*) *Plasmodiophora brassicae*, (*c*) *Saccharomyces cerevisiae*, (*d*) *Trypanosoma bruceri*, (*e*) *Crypthecodinium cohnii* and (*f*) *Oodinium, Amphidinium carterae*. For clarity, the outline of the cell has been omitted. Free microtubule-organizing centres in (*d*) and (*e*) correspond to basal bodies that are not involved in the set-up of the mitotic spindle. Numbered insets on the right-hand side are magnifications of the MT–kinetochore–chromosome interaction sites in the corresponding overviews on the left.

The most obvious route, and the one taken by higher eukaryotes, is to simply disassemble the nucleus at the onset of mitosis (prophase). This process, called NE breakdown (NEBD), begins with dispersal of nucleoporins, followed by the physical deformation and tearing of the NE by forces generated by dynein motors and microtubules, and finally the disassembly of the nuclear lamina, which is required for the structural integrity of the nucleus [29]. Concurrent with this process is the disassembly of the interphase microtubule cytoskeleton and the formation of asters around the two centrosomes, which then migrate to opposite sides of the nucleus. This migration is driven by molecular motors, which drive the extensile sliding of anti-parallel-oriented microtubules that originate from the two asters [30,31]. It is worth considering whether the two asters, connected by anti-parallel microtubule overlaps, constitute a bipolar spindle (rather than the textbook view that the bipolar spindle only assembles after NEBD). In fact, certain diatoms (e.g. *Lithodesmium undulatum*, *Surirella ovalis*) fully pre-assemble the spindle outside the nucleus. As NEBD occurs, the spindle sinks down into the chromosome mass [32–34]. In both diatoms and animals, the result is that the asters can access the chromosomal mass (and kinetochores), the mitotic spindle matures and the animal cell performs an ‘open’ mitosis (figure 2*a*).

## 5. Partial breakdown

The complete breakdown of the NE is, however, not always necessary. In principle, local openings within the NE (fenestrae), close to the MTOCs, are sufficient to allow MTs to capture the chromosomes; in *Caenorhabditis elegans* and syncytial *Drosophila melanogaster* embryos, for example, the mitotic spindle is established through polar fenestrae during prometaphase [35–37]. Unlike animal cells, NEBD in *C. elegans* is delayed until late anaphase. Interestingly, the multi-step process of NEBD (beginning with NPC removal) starts earlier in later embryo stages (from 30 cells, prometaphase) when compared with earlier (and smaller) embryo stages (2–24 cells, early anaphase) [37], suggesting that maintaining NE integrity for a longer time period provides benefits to smaller cell agglomerations. A variant of the semi-open mitosis that might represent an intermediate between semi-closed and fully closed mitosis can be found in *Plasmodiophora brassicae*, a unicellular plant pathogen causing root diseases in members of the Brassicaceae family. Electron microscopy (EM) reveals that the NE again disintegrates only around the centrioles, once they have fully separated during prophase [38]. The centrioles establish asters, and K-MTs radiate into the nucleus, where they capture the condensed chromosomes (figure 2*b*). Like animal cells, chromosomes attach to one or more spindle MTs via kinetochores, and EM data show that these kinetochores are bi-oriented [38]. Tension across sister kinetochores and the existence of a functional SAC are therefore possible. However, anti-parallel overlapping nK-MTs have not been described, suggesting that in anaphase chromosome segregation is driven by MT depolymerization and spindle elongation by aster-mediated cortical pulling forces only. The chromosomes do congress to the spindle equator, forming a metaphase plate, although the chromosomes are excluded from the centre of the plate owing to the positioning of the nucleolus. Chromosomes therefore adopt a ring-like conformation within the plate [38]. Whether the positioning of the nucleolus is a cause or consequence of this chromosome positioning is unknown. It is interesting to note that in human cells and mouse oocytes the chromosomes also adopt a radial organization early in mitosis that accelerates chromosome bi-orientation [39,40]. The ring-like metaphase plate in *P. brassicae* forces the spindle MTs into a cup-like structure, with the MTOC at its base and the chromosomes arranged around the opening. Interestingly, at the bottom of this cup-like arrangement, a remnant of the NE shields centrioles from the ‘nucleoplasm’ on axis with the nucleolus. Thus, the opening for the spindle MTs within the NE must have a ring-like shape with an NE-derived membrane centre. Membrane cisternae containing nuclear pores radiate along spindle MTs into the nucleus and also seem to be associated with chromatin, but EM data suggest that these cisternae are not continuously linked to the NE [38] (figure 2*b*, inset 2).

Like *P. brassicae*, the mitotic nuclei of the biflagellated algae *Chlamydomonas reinhardtii* open at the spindle poles into fenestrae, but unlike in *P. brassicae*, NE material is absent from the inner nucleus and the chromosomes. The flagella resolve during mitosis and allow the basal bodies to migrate towards the nucleus. At the end of this movement, basal bodies could be found proximal to at least one of the polar fenestrae [41,42]. The centrioles, however, seem to be dispensable for nucleation of spindle MTs [43,44]. Spindle MTs gain access to the chromosomes through the polar fenestrae and drive the formation of a metaphase plate [41]. Owing to weak contrast of MTs in the EM pictures provided in references [41,44], it remains unclear whether *C. reinhardtii* possess inter-polar microtubules and/or kinetochores. Thus, current data do not allow conclusions about the forces powering chromosome segregation and spindle elongation.

Although (partial) NE breakdown appears to be a straightforward solution that allows the MTOCs to gain access to the chromosomes, this approach might be hazardous as it exposes the genetic material to the cytosolic environment. Thus, especially for unicellular organisms, it might be favourable to maintain integrity of the NE during mitosis. On the other hand, maintaining the NE as a permeability barrier during mitosis could, in principle, allow for continuous transcription. Consequently, one element of the mitotic spindle (excluding the dynamic unstable MTs) has to span the NE in these organisms. Next, we present examples for organisms that have developed either membrane-based MTOCs or kinetochores that allow a fully closed mitosis.

## 6. Nuclear-membrane-based microtubule-organizing centres

Prominent and well-described examples of nuclear-membrane-based MTOCs can be found in the fungal Ascomycetes phylum: the MTOCs (spindle pole bodies, SPBs) of *Schizosaccharomyces pombe* can be regarded as a further functional step from partial open mitosis towards consequent closed mitosis. Its SPBs duplicate on the outer surface of the NE and are transiently inserted into nuclear fenestrae, which close again after anaphase onset and extrude the SPBs back into the cytoplasm [45]. By contrast, the composition of *Saccharomyces cerevisiae* SPBs represents an MTOC configuration fully adapted to permanent insertion into the NE. It consists of three distinct layers: a crystalline central protein plaque residing in the plane of the NE and attached to it; and an inner and outer plaque, both independently organizing microtubules in the nucleus and the cytoplasm, respectively (figure 2*c*). For a comprehensive overview of fungi SPBs, we would like to refer to the review by Jaspersen & Winey [46]. Although best studied in fungi, membrane-based MTOCs are not restricted to them. Microtubule-organizing activity in the cytoplasm and nucleus of the flagellated unicellular parasite *Trypanosoma brucei* is clearly separated. Centrioles of the basal body organize the flagellum and the distribution of the kinetoplast (a single large mitochondrion, containing a complex network of mitochondrial DNA) during closed mitosis [47]. Separate EM-evident MTOC structures within the NE organize the mitotic spindle. Like *P. brassicae* (see above), spindle MTs are aligned around the nucleolus, which stretches from pole to pole during metaphase [48,49]. Unlike yeast SPBs, the MTOCs of *T. brucei* lack any sub-layer on the cytoplasmic face of the NE and consequently do not nucleate any astral microtubules [48]. To date, a biochemical or structural analysis of these structures is missing (figure 2*d*).

As expected, *T. brucei* [48] and other species that use NE-based MTOCs have classical ‘animal-like’ kinetochores that allow for bi-orientation and the possibility of a tension-dependent SAC. Kinetochore-independent segregation mechanisms are also possible and have been discussed for *T. brucei* because the large number of chromosomes (approx. 100 mini-chromosomes and approx. 20 large chromosomes [50,51]) does not match the small number of visible kinetochores (approx. 8) [48] and spindle MTs (approx. 20) [49]. However, the fact that the chromosomes of *T. brucei* do not condensate [52] during mitosis makes an EM-based assignment of kinetochores to single chromosomes difficult. This led to the proposal that several chromosomes share one kinetochore structure [48]. Nevertheless, mini-chromosomes do congress to the spindle equator and are segregated synchronously in an MT-dependent manner [53].

## 7. Nuclear-membrane-based kinetochores

Like yeast, members of the phylum Dinoflagellata and the class of Parabasalia (from the Protista kingdom) also conduct a closed mitosis. Dinoflagellates are unicellular, flagellated organisms that are mainly free-swimming in the sea as part of the plankton, although fresh-water and parasitic (*Oodinium* [54]) species have also been described [55]. Parabasalids are anaerobic multi-flagellated organisms that are obligatory, symbiotic or parasitic, and can be found in the digestive tracts of organisms such as termites and cockroaches. Amazingly, these species actually insert their chromosome–microtubule interface into the NE. This implies that these organisms have to assemble the mitotic spindle outside the nucleus. In the case of the dinoflagellates, this has led to a rather unconventional spindle set-up: in *Crypthecodinium cohnii*, the bipolar spindle is organized by two acentriolar MTOCs that are located in the cytoplasm on either side of the nucleus [56]. Unlike commonly known nuclei, those in mitotic dinoflagelates possess tunnels (called channels) that span the nucleus from one MTOC to the other (figure 2*e*). Spindle MTs pass through these channels, resulting in a weakly focused spindle without asters [56,57]. The chromosomes of *C. cohnii* are attached to the inside of the nuclear membrane. This is opposite the position within the nuclear channels where spindle MTs terminate [58,59] (figure 2*e*). Thus, chromosomes and microtubules are physically separated by the NE. The membrane geometry of these attachment sites suggests that MTs are attached end-on, and the concepts of bi-orientation and tension generation might apply (figure 2, inset 3). However, early studies assumed that chromosomes attach to the lattice of the microtubules, and proposed that membrane synthesis drives chromosome separation towards the poles along the spindle exoskeleton [57]. In terms of kinetochores, the literature has been indecisive: Bhaud *et al.* [59] described a membrane thickening at the attachment sites that might harbour transmembrane elements that link chromosomes and microtubules. They could not, however, detect any classical kinetochore-like structure. By contrast, earlier EM data show ill-defined electron-dense material on both membrane faces of the attachment site, arguing for the existence of kinetochores [57,58]. The end-on nature of these attachments could allow the generation of pulling forces through MT depolymerization.

**Figure 3.**
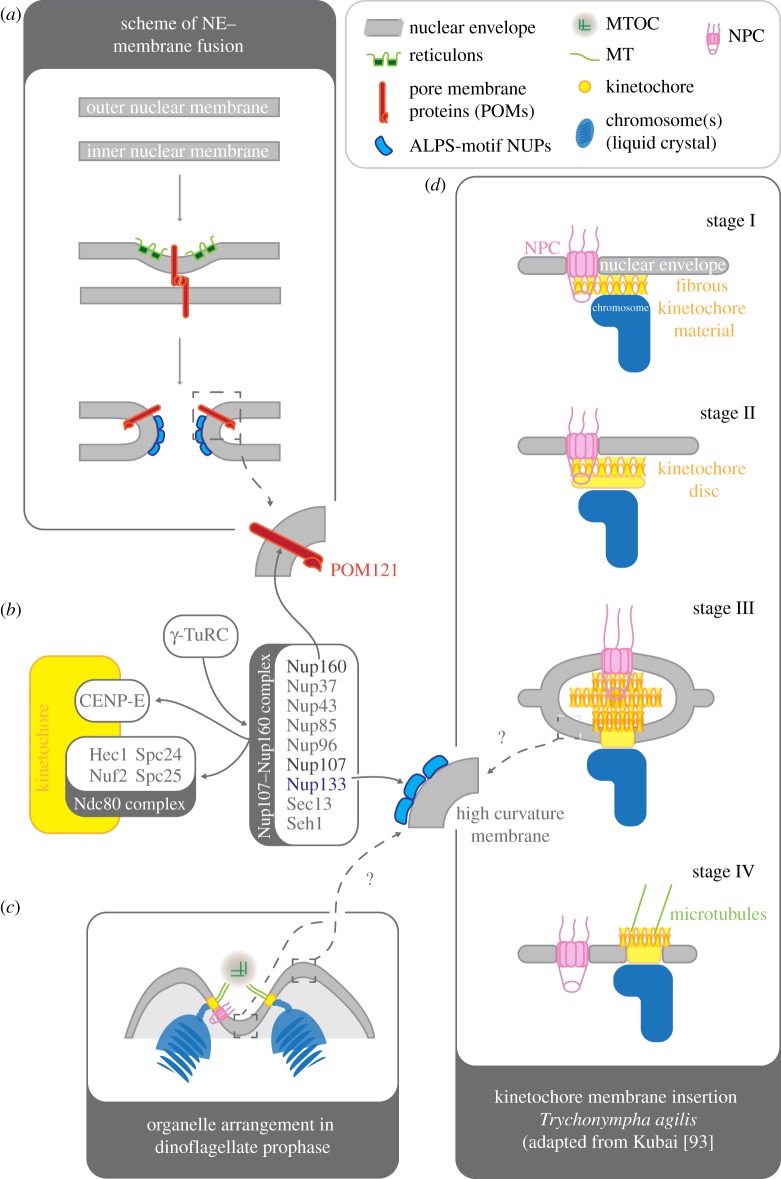
Hypothetical overview of interactions and mechanisms that might mediate the insertion of membrane-bound kinetochores into the nuclear envelope (NE). (*a*) Summary of the mechanisms that lead to *de novo* insertion of nuclear pore complexes (and spindle pole bodies) into the NE. Collaborative action of transmembrane pore membrane proteins (POMs) (red) and reticulons (green) results in the convergence of the inner and outer nuclear membrane, leading to the eventual fusion of membranes. The result is a pore in the NE. Once the pore has established POMs and nucleoporins (NUPs) containing ALPS-motifs (see main text) line the highly curved membrane within the nuclear pore and provide a membrane-scaffold for further members of the nuclear pore complex (NPC) to assemble on. (*b*) The Nup107/160 complex is a central hub that links to the kinetochore and might mediate membrane insertion of kinetochores. Nup107/160 interacts with CENP-E and the Ndc80-complex at the kinetochore, as well as with the gamma tubulin ring complex (γ-TuRC)—a ring-like matrix that contributes to MT-nucleation. Nup160 was shown to bind to POM121 within the membranes around the nuclear pore. Nup133 was shown to contain a functional ALPS-motif (see text) that specifically binds to membranes of high curvature and is thus able to directly interact with the membrane environment within the pore. (*c*) Diagram of a prophase arrangement in (some) dinoflagellates in which kinetochores are inserted in the NE. The MTOC(s) have invaginated into the nucleus. Chromosomes cluster by this invagination, which is characterized by a high density of nuclear pore complexes and membranes with high curvatures. (*d*) Kinetochore maturing and membrane insertion in *Trychonympha agilis* as described by Kubai [93]. Stage I: fibrous kinetochore material, associated with a NPC, starts to assemble between the chromosome and the NE. Stage II: the kinetochore matures as a dense disc positioned between chromosome and the fibrous material. Stage III: an intermediate structure within the insertion process, resembling an intra-membrane vesicle. The dense kinetochore disc has already been inserted into the NE. The vesicle structure is filled with the filamentous material and contains at least one NPC. Stage IV: the vesicle structure has resolved. The fibrous kinetochore material is now on the cytoplasmic surface of the NE and able to accept spindle MTs.

In other dinoflagellates (i.e. *Oodinium*, *Amphidinium carterae* and *Syndinium*), a single central channel spans the nucleus from pole to pole, harbouring nK-MTs and K-MTs [54,60,61] (figure 2*e*). EM stills from different times in the cell cycle suggest that this central channel starts as an NE invagination around the MTOCs that eventually pierces through the nucleus during spindle pole separation, leaving one central channel [54,61]. As soon as the duplicated MTOCs invaginate into the NE the chromosomes become clustered proximal to the MTOCs (figure 3*c*). EM data reveal an electron-dense material at the attachment sites of chromosomes and MTs [54,61]. These structures show a fibrous corona towards the end-on-attached MTs and have been described as a bilayered structure—one layer associated with the chromosome, the other with the microtubule itself–thus showing properties of a classical kinetochore [61]. In the presented sections, kinetochores of *Oodinium* and *A. carterae* are occupied by one or two microtubules only [54,60], whereas kinetochores of *Syndinium* are attached to multiple MTs [61]. Once spindle pole separation has occurred, and the central channel has been established, sister chromosomes in *Oodinium* are aligned along the central channel and seem to be bi-oriented as proximal kinetochores are diametrically attached to K-MTs emanating from opposite poles (figure 2*f*). Geometry of the surrounding membrane (i.e. a 45–90° angle between channel axis and kinetochore plate) further suggests that these kinetochore pairs experience tension [54] (figure 2, inset 3). Because nK-MTs span the central channel, both depolymerization of kinetochores MTs and motor-driven lateral sliding of K-MTs along nK-MTs could empower tension generation and polewards chromosome movement in anaphase. Interestingly, this mechanism of chromosome segregation is not exclusive to protista/dinoflagellates. For example, *Ostreococcus tauri*, an unflagellated algae described to be the smallest eukaryote yet discovered [62], establishes a mitotic spindle through a single nuclear spindle tunnel and uses microtubule-dependent chromosome segregation [63]. Hence, we speculate that this organism also uses membrane-based kinetochores. Although no kinetochores have been described by EM, classical kinetochore components such as *Ndc80*, *Nuf2*, *CENP-C* and SAC proteins (*Mad1*, *Mad2*, *Cdc20*, *Bub1*) have been identified in its genome [64].

We have reasoned that membrane-based kinetochores are a hallmark of dinoflagellate mitosis. Most dinoflagellates, such as *C. cohnii*, contain uniquely compacted chromosomes in that DNA is condensed throughout the cell cycle and associated to the NE. With exceptions such as *Syndinium*, DNA condensation is not achieved by ‘conventional’ nucleosomes packaging [65], but by the formation of liquid crystal structures [59,66–68]. These chromosomes contain a low protein content, dominated by a few dinoflagellate-specific, basic histone-like proteins (HLPs) [69] that locate to the chromosome periphery and non-crystalline, transcriptionally active loops [70–72]. One could now reason that a liquid crystal must demand a specialized protein–DNA (crystal) interface, arguing that dinoflagellate membrane-bound kinetochores might significantly differ from those of *O. tauri* or other ‘classical’ kinetochores. However, EM data show that DNA–membrane attachments are mediated by decondensed loops of the chromosome [58,59]. Thus, described HLPs might serve as scaffold for a kinetochore structure. More likely, though, dinoflagellates also might use the ‘classical’ kinetochore DNA-binding interface, as a recent analysis of dinoflagellate expressed sequence tag (EST) libraries reveals the existence of conventional histone H2B, H3, H4 and the H2A variant H2A.X [73] (see also [74] for a comprehensive review on this topic). This would suggest that their kinetochores differ from their animal counterparts only in the transmembrane part.

## 8. Using existing infrastructure? Nuclear pores and membrane-based kinetochores

In the context of nuclear-membrane-based kinetochores, the interesting question remains as to how new kinetochores insert into the membrane. Given the limited data on this process, it is worth summarizing the current view on how MTOCs (i.e. SPBs in yeast; for a detailed review, see [75]) are inserted into the NE. In budding yeast, the SPB duplicates on the cytosolic surface of the NE and has to be inserted into the NE across both the inner and the outer membranes. It emerged that NPC and SPB insertion requires the same NE modification steps that eventually end in the fusion of outer and inner nuclear membrane, leaving an opening in the NE. Fusion starts with constriction of the inner and outer NE bi-layer, thereby narrowing the perinuclear space (figure 3*a*). The membrane constriction is probably mediated by protein–protein interactions between perinuclear parts of transmembrane proteins (POMs) in both bi-layers. ER-derived intra-membrane proteins called reticulons are thought to stabilize the resulting curvature of the membrane by interleaving into the outer lipid layer [28,75,76]. Following nuclear membrane fusion, POMs are thought to mediate the attachment of the nuclear pore and SPB complexes to the membrane (figure 3*a*). In *S. cerevisiae*, the POM Ndc1 has been reported to take part in both SPB and NPC insertion, and tethering in the NE [77–80]. However, while Ndc1 is essential to SPB insertion, its role in NPC insertion seems to be redundant with POM152 [79,81]. By contrast, POMs seem to be dispensable for the insertion of NPC into the NE in *A. nidulans*—as long as they express a functional Nup84/120 complex [28,82]. This points to an alternative mechanism that associates nucleoporins with the NE [83].

In this regard, *S. cerevisiae* Nup85 and Nup120 (both part of the fungal Nup84/120 complex), as well as human Nup133 [84], are predicted to contain an ArfGAP1 lipid packing sensor (ALPS) motif, which has shown to be a membrane-binding, amphipathic alpha-helix [84,85]. The ALPS motif selectively binds membranes of a high curvature, presumably by interleaving hydrophobic residues in between exposed lipid molecules on the convex site of the curvature [85]. Thus, these motifs have been proposed to mediate nucleoporin interaction with the highly curved membrane around the insertion site of the NPC complex [28,75,84] (figure 3*a*,*b*). Interestingly, human Nup133 is a subunit of the Nup107/160 complex (figure 3*b*), which, besides being a structural component of the NPC, is also important during mitosis, when it transiently localizes to chromatin and kinetochores [86,87]. Selective removal of the kinetochore-bound pool of the complex affects recruitment of RanGAP/CRM1 [88], the chromosomal passenger complex [89] and the microtubule-nucleating gamma tubulin ring complex [90], resulting in pleiotropic effects on chromosome segregation (for comprehensive reviews, see [88,89,91,92]). With regard to membrane-based kinetochores, we would like to emphasize that kinetochore localization of Nup107/Nup160 is mediated by the outer kinetochore components CENP-F and the Ndc80-complex [88] (figure 3*b*). We hypothesize that in protists, which use membrane-based kinetochores, a conserved interaction between the outer kinetochore and a Nup107/Nup160-like complex could mediate the insertion of the kinetochore into the NE via ALPS-motif-containing nucleoporins. Alternative mechanisms, analogous to those involved in SPB/NPC insertion (i.e. usage of POMs and reticulons), are also possible (figure 3).

In either case, NPC structures have to be shared. Hence, insertion of kinetochore discs into the NE should occur proximal to an already inserted NPC, as during SPB insertion. This is indeed the case in *Oodinium* and *Syndinium*, where (new) kinetochores are always established adjacent to nuclear pores [54,61] (figure 3*c*). Furthermore, *Oodinium* kinetochores are thought to mature during mitosis [54], a view that is strengthened by ultra-structural observations on the closed mitosis of the parabasalid *Trychonympha agilis*. In contrast to the dinoflagellates, *T. agilis* does not generate a nuclear channel, but builds a ‘conventional’ exospindle that laterally attaches to the nucleus [93]. Nevertheless, *T. agilis* connects its spindle to chromosomes via membrane-based kinetochores. Kubai [93] described a possible mechanism (figure 3*d*), in which kinetochores assemble between chromatin and the inner nuclear membrane on a precursor that is associated to nuclear pores throughout interphase. Ultra-structural data suggest that the kinetochore gets inserted from inside the nucleus into the NE, passing through a putative transient state that resembles an intra-membrane vesicle structure within the NE. This structure contains the already inserted kinetochore disc, as well as nuclear pores, and is made of curved membranes, allowing speculation that this yet uncharacterized mechanism of kinetochore insertion might use ALPS-motifs (figure 3). Although this review does not reveal how these structures are established or resolved, it suggests that nuclear pores play an important role in the process of kinetochore insertion.

Interestingly, a more precise look at the EM data of kinetochores in the aforementioned *P. brassicae* (see §5) also shows an intimate relation between nuclear membrane and kinetochore structures. Although *P. brassica* conducts a partial open mitosis, it also seems to use membrane-associated kinetochores. Associated membrane material is evident in nearly all kinetochore close-ups provided. Especially, figure 23*b* of [38] (figure 2, inset 2) shows that the thickness of the kinetochores nicely correlates with that of the membrane attached to it. Although these electron micrographs do not show clear evidence of adjacent nuclear pores, the authors report that this membrane material contained NPCs. In summary, it is plausible that the insertion of kinetochores and SPBs evolved using an existing intra-membrane structure, the nuclear pore, as an entry vector into the NE.

## 9. Architecture of a membrane-based kinetochore

Membrane-based kinetochores must feature a transmembrane element, not found in classical kinetochores. This element could range from a specialized intra-(trans)-membrane protein that independently recruits an MT-binding interface on the outer face and a DNA-binding interface on the inner NE-surface, to a compact kinetochore complex that fully penetrates the NE. In either case, conventional models of kinetochore-centric mechanisms will be challenged: if both binding interfaces can be recruited independently, how would simultaneous attachment be coordinated, and how could there be any communication between cytosol and nucleus? Observations made in *C. cohnii* suggest that this organism possesses an APC-dependent SAC [94]. Some communication must therefore take place between compartments. Furthermore, chromosomes seem to be bi-oriented in dinoflagellates (see §7). How could an Aurora-B-based tension-sensing mechanism [21,95], thought to depend on mechanical deformations within the kinetochores complex, work across the NE? Hence, we would favour the existence of a compact kinetochore complex that spans the NE. So, what would the central, intra-membrane part look like? Would it be analogous to the crystalline central plaque of yeast SPBs or could it use subcomponents of NPCs? Owing to the close linkage between the NPC and kinetochore/SAC components (see §8), and the fact that tension sensing by mechanical deformation seems to be incompatible with a crystalline central layer, we would hypothesize that the central layer of membrane-based kinetochores will be related to NPC components. Future investigations into the structure and composition of the transmembrane element, as well as the mechanism of (*de novo*) insertion into the membrane, should provide new insights into how large protein complexes (such as the NPC and SPB) are inserted into the NE.

## 10. Future perspectives

In this review, we focused on a major challenge during chromosome segregation: to join cytoplasmic MTOCs and nuclear chromosomes, spatially separated by the NE. We highlighted solutions that are significantly different from those that have intensively been studied in human, mouse, fly and yeast. We would like to encourage (re-)examination of mitosis and chromosome segregation in these non-model organisms for the following reasons. First, particular model organisms have been chosen for different reasons; once for their relevance (human cell culture), but mostly just for practical issues such as small genome size, feasibility of genomic manipulation, ability to culture the organism in the laboratory and so on. This does not necessarily mean that the studied organism is representative of the majority of its species; it could as well be the exception, not the rule. Second, a comparison of different evolutionary approaches to the same problem might reveal a conserved, basic mechanism. Third, most the work on the non-model organisms that we describe in this review is based on pioneering use of thin section negative stain electron microscopy mainly generated during the 1970s. These allow insights into the ultra-structure of spindle apparatus geometry as well as the topography of MTOCs and/or kinetochores. However, these snapshots of fixed cells are not adequate to decipher complex dynamic cell biological processes such as mitosis. We believe that the application of contemporary cryo-EM tomography on these organisms might improve the spatial resolution of crucial structures such as MTOCs or kinetochores and reveal information on the mechanism that inserts them into the NE. In the recent past, methods have been established in yeast that allow purification of kinetochore sub-complexes, or even complete kinetochores [96–101]. Successful adaption of these methods to dinoflagellates could allow us to identify the nature of membrane-based kinetochores and how they differ from free ‘classical’ ones. This can be backed up by next-generation sequencing and bioinformatics approaches to search for protein-orthologues of known kinetochore components in the dinoflagellate genome. The huge number and the crystalline nature of dinoflagellate chromosomes might hinder this approach at the DNA level, but the feasibility to identify homologues from EST libraries has been proved [68]. Finally, the development of methods to genetically modify these non-model organisms will allow us to use fluorescent proteins and live-cell microscopy to provide the first complete dynamic description of these exotic mitotic mechanisms.

## 11. Acknowledgements

We apologize to colleagues whose work could not be cited owing to space constraints. We thank Masanori Mishima, Jonathan Millar, Anne Straube and Dimitris Liakopoulos for helpful discussion and comments on the manuscript, as well as Elina Vladimirou for critical reading. This work was supported by research grants from Marie Curie Cancer Care and the BBSRC (BB/I021353/1).
